# Frequency of Toxoplasmosis in Water Buffalo (*Bubalus bubalis*) in Trinidad

**DOI:** 10.4061/2011/705358

**Published:** 2011-11-28

**Authors:** Anil Persad, Roxanne Charles, Abiodun A. Adesiyun

**Affiliations:** School of Veterinary Medicine, Faculty of Medical Sciences, The University of the West Indies, St. Augustine, Trinidad and Tobago

## Abstract

Toxoplasmosis has been reported to occur in several animals and humans causing different clinical manifestations. The study was conducted to determine the frequency of *Toxoplasma gondii* antibodies (IgG) in water buffalo (*Bubalus bubalis*) across farms in Trinidad using a latex agglutination test. Of a total of 333 water buffalo tested, 26 (7.8%) were seropositive for *T. gondii* antibodies. Seropositivity for toxoplasmosis was statistically significantly (*P* < 0.05; *χ* ^2^) higher in adult water buffalo, 12.4% (14 of 113) compared with young water buffalo, 4.2% (6 of 143). Seropositivity for toxoplasmosis across the seven farms ranged from 0.0% (0 of 20) in Farm G compared with 20.0% (10 of 50) detected in Farm B. The differences in seropositivity by management system, free-ranging 6.7% (14 of 213) and semi-intensive 10.0% (12 of 120) and by sex, in male 6.7% (7 of 104) and female 8.3% (19 of 229) water buffalo, were not statistically significant (*P* > 0.05; *χ* ^2^). This is the first documentation of toxoplasmosis in water buffalo in Trinidad.

## 1. Introduction

Toxoplasmosis is an important protozoan parasitic zoonosis which has been documented in a wide variety of animals (domestic and wild) and in humans, occurring in both clinical and subclinical forms [1, 2]. Felidae are considered the primary reservoir for toxoplasmosis [1], and food animals have been established as sources of infection for humans through exposure to the bradyzoites and tachyzoites [2]. Foods such as vegetables have been documented to be contaminated by the oocysts of *T. gondii* and causing toxoplasmosis in human consumers [3]. Consumption of poorly cooked meat and meat products containing the parasite has been reported to be one of the routes of human exposure and toxoplasmosis [3]. Several risk factors have been reported for toxoplasmosis in humans and livestock [1, 4].

Serological studies on toxoplasmosis have been reported in water buffalo elsewhere, where seropositivity ranged from 0%–8.8% [5–7]. Several serological tests have been used to detect toxoplasmosis including the enzyme-linked immunosorbent assay (ELISA) [8], indirect fluorescent antibody test, IFAT [5, 8], capillary agglutination test [9], and latex agglutination test [6, 8] with varying sensitivity and specificity. 

In Trinidad and Tobago, toxoplasmosis has been reported in humans including apparently healthy sugarcane field workers [10], dogs [11], livestock [9], and goats experiencing abortions in Tobago [12]. 

To date, there is a dearth of information on the frequency of toxoplasmosis in water buffalo in Trinidad and Tobago. The study was conducted to determine the seroprevalence of toxoplasmosis in water buffalo and to relate infection to age, sex, management system, and farms in different locations in Trinidad.

## 2. Materials and Methods

### 2.1. Water Buffalo Farming in Trinidad

Water buffalo production in Trinidad and Tobago is distributed among a few large farms primarily owned by companies and several hundred small holdings located mainly in the sugarcane-growing areas of the country [13]. Water buffalo are typically raised under extensive management conditions, and farmers, excluding the large farms, generally own only 2–5 head. At the time of the study, the water buffalo population in Trinidad was estimated to be 5000 [14]. Water buffalo are raised mainly as beef animals while a few owners use them as draught animals for the transportation of harvested sugarcane in carts.

### 2.2. Sources of Water Buffalo Serum Samples

Serum samples used in this study were collected during an investigation on brucellosis in water buffalo and cattle in Trinidad [15, 16] over a two-month period. The sera were thawed out only twice for both studies on brucellosis prior to the current study. All sera were thereafter stored at −20°C prior to being screened for toxoplasmosis in the current study. Only sera from water buffalo farms with animal populations exceeding 150 animals were included in the current study. For this investigation the farms were described as extensively managed when the water buffaloes were always on the pasture and semi-intensively managed when the animals were on the pasture during the day but returned to the paddocks or pens during the night. 

### 2.3. Sample Size Determination

A calculated sample size of 250 was determined using the formula: estimated sample size (*n*) = 1.962 × *p*(1 − *p*)/*L* ^2^ [17] where *p* = reported prevalence of 3% for toxoplasmosis in water buffalo [18] and a desired precision of 2%. However, considering the number of samples the test kits could perform, a total of 333 samples were tested.

### 2.4. Selection of Samples for Testing

Using the pool of serum samples from the seven farms and considering the total number of samples, through proportional representation based on the total number of sera available per farm, the number of samples to be tested from each of the seven farms (Table 1) was randomly selected from the pool.

### 2.5. Detection of *T. gondii* Antibodies

The latex agglutination test (Toxotest—MT “Eiken,” Japan) with a sensitivity of 99% and a specificity of 81%, as stated by the manufacturer, was used to detect antibodies to *T. gondii*. Initially, all serum samples were screened at a titre of 1 : 16 and all positive samples were subsequently tested at dilutions of 1 : 32, 1 : 64, 1 : 128, and at 1 : 256 with concurrent testing of positive and negative controls. For this study a cutoff titre of 1 : 64 or greater was classified as positive as recommended by the manufacturer and as earlier reported [11].

### 2.6. Statistical Analysis

The frequency of detection of antibodies to *T. gondii* was compared for age, sex, management system and location of farms after processing the data using the Statistical Package for Social Sciences (SPSS) version 10. The chi-square test was used to determine statistically significant differences between the frequencies using alpha at 0.05.

## 3. Results

### 3.1. Seropositivity for Toxoplasmosis in Water Buffalo

Overall, of 333 water buffalo tested, 26 (7.8%) were seropositive for *T. gondii* antibodies with titres of 1 : 64 or greater. Of the seropositive samples 12 (3.6%) and 3 (0.9%) of 333 water buffalo tested had titres of 1 : 128 and 1 : 256, respectively.

### 3.2. Seropositivity for Toxoplasmosis by Risk Factors

 Seropositivity for toxoplamosis was statistically significantly (*P* < 0.05; *χ* ^2^) higher in adult water buffalo (12.4%) compared with young water buffalo (4.2%) as shown in Table 1. Similarly, seropositivity for toxoplasmosis was significantly (*P* < 0.05; *χ* ^2^) different for water buffalo farms with a range from 0.0% (0 of 20) on Farm G compared with 20.0% (10 of 50) on Farm B. Neither the sex of animals, male (6.7%) compared with female (8.3%) nor the type of management system, free-ranging (6.7%) versus semi-intensive (10.0%), has a significant (*P* > 0.05; *χ* ^2^) effect on seropositivity.

## 4. Discussion

The seropositivity of 7.8% for toxoplasmosis in water buffalo detected in the current study is double the rate reported for water buffalo in Brazil also using the latex agglutination test and a cutoff titre of 1 : 64 [6]. It is therefore evident that exposure experience for toxoplasmosis in water buffalo in Trinidad is higher than in Brazil. Other researchers have reported varying rates of seropositivity for toxoplasmosis in water buffalo in several countries including India, 2.9% using ELISA [19], China and Egypt (both 0.0%) using indirect agglutination test [7, 20]. South Vietnam, 3.0% with the direct agglutination test [18], 8.8% in Iran using the IFAT [5], and 20.4% in Afghanistan as detected by the micromodification of indirect haemagglutination test [21]. It is, however, pertinent to mention that it has been documented that the sensitivity and specificity of serological tests used for the detection of *T. gondii* affect the seropositivity rates detected [22].

The fact that water buffalo in Trinidad are predominantly reared as beef animals coupled with the finding that 7.8% were seropositive for toxoplasmosis suggest that meat from slaughtered parasitized water buffalo may serve as a source of exposure for humans who consume improperly cooked meat. The risk appears real due to the fact that meat from water buffalo is frequently sold to unsuspecting consumers as beef and therefore exposure to toxoplasmosis following consumption of meat from water buffalo is possible. Consumption of improperly cooked meat from livestock has been associated with infection by *T. gondii* and clinical toxoplasmosis in humans [1, 3]. In Trinidad and Tobago, serological evidence of toxoplasmosis exist in cattle, pig, goat and sheep [9], and in humans [10].

The age of animals was significantly associated with seropositivity for toxoplasmosis detected as older water buffalo were more infected than young animals. This finding could be explained, in part, by the fact that with increase in age, exposure to toxoplasmosis was expected to be higher. Toxoplasmosis was found to occur at a higher frequency in older than younger animal species by others in Nigeria and Mexico [23, 24]. However, Navidpour and Hoghooghi-Rad [5] found the exact opposite result in a study in Iran where a significantly higher seroprevalence of toxoplasmosis was detected in water buffalo less than 1 year old (10.8%) than in those 1 year and older (4.7%). The differences observed between both studies may reflect differences in management practices used for water buffalo in Trinidad and Tobago and Iran. It has been documented that exposure to cats and their oocysts in the environment may affect exposure to *T. gondii* by livestock [1]. 

The seropositivity of toxoplasmosis in water buffalo across the seven farms in the country were found to be significantly different, a finding which may reflect varying exposure to the oocysts of *T. gondii,* primarily from cats and also to wild rodents in the pasture [1, 25].

It was surprising to detect that the seropositivity for toxoplasmosis on water buffalo farms that were free-ranging or extensively managed and semi-intensively managed did not differ significantly. It is expected that exposure to cats and their faeces will be higher for semi-intensively managed water buffalo compared with those free-ranging or extensively managed farms. 

The seropositivity of toxoplasmosis in male and female water buffalo were similar, an indication that the sex of the animals did not significantly affect exposure to toxoplasmosis. Navidpour and Hoghooghi-Rad [5] had also reported that sex was not an important factor in the seropositivity of water buffalo in Iran to toxoplasmosis as was also detected in other livestock [25, 26].

## 5. Conclusions

 It was concluded that toxoplasmosis exists in water buffalo population on large farms in Trinidad and Tobago, a finding being documented for the first time on six of the seven farms studied. The fact that water buffalo meat is frequently sold as beef to the public means that improperly cooked water buffalo meat from seropositive animals may serve as sources of *T. gondii *in consumers.

## Figures and Tables

**Table 1:** Frequency of toxoplasmosis in water buffalo by risk factor.

Parameter	No. of animals tested	No. of (%) seropositive
Age^a^		
Young	143	6 (4.2)
Adult	113	14 (12.4)
Unknown	77	6 (7.8)

Sex		
Male	104	7 (6.7)
Female	229	19 (8.3)

Management system		
Free-range	213	14 (6.7)
Semi-intensive	120	12 (10.0)

Farm location		
A	50	2 (4.0)
B	50	10 (20.0)
C	50	4 (8.0)
D	13	1 (7.7)
E	75	6 (8.0)
F	75	3 (4.0)
G	20	0 (0.0)

^a^<2 years old were considered young and >2 years old were classified as old.
